# Gay Life in Paris and the Water Cure in Germany1Correspondence from Louisville Monthly Journal of Medicine and Surgery.

**Published:** 1909-10

**Authors:** J. M. Mathews

**Affiliations:** Wiesbaden, Germany


### Gay Life in Paris and the Water Cure in Germany.[1]

*To the Editor Louisville Monthly Journal:*

You will, I know, permit me to give your numerous readers my impression of some few places that I have seen and the people thereof in this short run through a portion of Europe. Individual impressions are of but little real value, I know, for one person may go into ecstacy over a certain picture or a piece of sculpture that another sees no beauty in at all. So it is, too, with the observation of places, be it the natural beauty of Versailles or the ponderous structure of London—one person preferring the former, the other the latter.

But of one place all are agreed, the Mecca of all tourists, is Paris—dear, sweet, charming Paris. It cannot be compared to

1. Correspondence from Louisville Monthly Journal of Medicine and Surgery.

any other city or place, for the comparison would indeed be odious. Call its life froth, foam or whatever you will, you will beg for more of it. Call its people effervescent, gushing, deceitful if you will, but you are bewitched and entranced by them.

THE PALM AND SILVER.

You may recognise that the hearty shake of the hand means only the extraction of francs from your palm, but you enjoy it. When in its atmosphere one thinks only of the perfume of its flowers, the grandeur of its art, the exhilaration given by its sparkling wine, and the soothing effect of its music. If you are so constituted that these things do not move or concern you, then listen to the chimes of the bells in the towers of Notre Dame de Lorette, or view the Madeleine, the most beautiful edifice in Paris, or the Pantheon, and hover over the tombs of Voltaire, Rousseau and other noted men.

If of a morose nature, go to the Place Vendome and mingle with the mob and follow it to the Place de la Concorde and watch them bring Marie Antoinette from the prison of the Conciergerie and cut off her head. If not yet satisfied, go over to the tomb that holds the bones of the "Little Corporal" and look down on the man that Bob Ingersoll said had caused more human sobs and tears than any other person who had ever lived. But beware, for though dead he liveth, and his very presence is felt as you stand under the great dome that shelters his remains, for truly he was the greatest warrior that ever lived or died.

MAXIM'S AT MIDNIGHT.

But better it would be if you would wander through the Louvre, take a drive through the Bois de Boulogne, dine at Henry's, the oldest, yet the best cafe in Paris, visit the grand opera, and at the stroke of 12, midnight, go into Maxim's and enjoy life with the boys and girls. And so it is that you say on leaving the charming city, not good-bye, but au revoir, for sure you expect to come again, for if you do not you are a greater sinner than any that you met within its portals.

THE WOMEN OF PARIS.

This note of observation would be incomplete if I did not mention the women of Paris—indeed, nothing is complete without a woman. Well, a critic must be both fair, just and truthful. These dear creatures must be compared to the girl from ——— who landed in New York on a Monday night and wrote back to her girl friend at home: "What a pity that it is not Saturday that I could take a bath, for the bathroom is a beauty bright." The bathrooms of Paris are few and far between, so no wonder that even these fair and lovely creatures are deprived of that great luxury, the daily bath. But their gowns, which are exquisite, seem to fit them to perfection, and the chic is purely French. As to beauty—Louisville with its population of three hundred thousand can produce more beautiful women to the square foot than can this great metropolis with its population of three million souls. So say we all.

### SPRING WATER LAND.

A rapid express train leaves Paris every morning at 9 o'clock for a twelve-hour run to Mayence, Germany. This train I took. Of all countries on the globe Germany boasts of more mineral springs—good ones, too—than can be found in any other. Three hours after our train started it came to a halt and the passengers were requested to alight and drink freely of the waters of a spring hard by said to possess great medicinal virtues. Four hours afterward we passed Worms, the quaint old city made famous by Martin Luther. Many other cities of note lie nestling in the hills along this route. At 9 o'clock p. m. Mayence was reached and in stepping out we felt that we were on solid earth again—no froth, no foam, but in lieu thereof good and honest feelings, such as pervade the true German character always and everywhere. Even the very handshake carries with it sincerity. No duplicity here; ask for what you want and you get it, and that too without feeling that you have violated any hard-set rule.

### GERMAN HOSPITALITY.

The stranger when within their gates is no longer a stranger, but is made to feel at home. A few days ago the assistant postman left his office bareheaded, and went with me a full half square to show me the direction I sought. How it reminded me of dear old New Orleans, where one will cease his work to do a stranger a service.

Talk of hospitality, I want none purer than is found here in Germany. It is only a fifteen-minute ride by electric or steam cars to Wiesbaden, the place from which I am now writing. It is beyond doubt the most beautiful city in Germany, if not in Europe. It claims a population of 150,000, but, although this is off season, there are 80,000 visitors here. What, with its parks, lakes, fountains, statues, music, flowers and handsome Kursaal, there is nothing left to be desired.

I have never been impressed with the hotels in Europe, but an exception must be made here. No more luxurious or handsomer ones can be found than are in Wiesbaden. The one at which I am stopping covers one whole square, yet it cannot accommodate any more guests than can our Seelbach, being only five stories in height. Wiesbaden is admittedly the queen of continental spas, not to speak in any disparagement of other noted ones, such as Carlsbad, Baden-Baden, etc.

PLACE FOR CURE.

As a medical man I would advise those seeking relief from the many ills that flesh is heir to, to come here rather than go to the others. The climate is warmer and more equable, and the waters equally efficacious, and the expense not near so much. The great spring, Kochbrunnen, is a seething, boiling water, pouring up and out of the earth. Because of the heat of its water it takes fully ten minutes to sip a glass of it. There are many bathhouses, but the main one is a wonder in extent and luxuriousness. An inspection of all persons desiring the baths is expected to be made by a competent medical man, for which a reasonable fee is charged, both for baths and advice. You would have to pay both, but I, as Senator Bailey would say, am exempt from such, as I found on my desk a card addressed to Herr Professor Doctor Med. Mathews, admitting me to all the privileges at Wiesbaden. So much for being a medical man.

HARD PLACE TO LEAVE.

One lingers here long after a determination to go, and then it is with deep regret. My intention in writing was only to give my impressions of the two places—Paris and Wiesbaden, and draw a comparison if I could of the two peoples—German and French. Therefore, to summarise: The French are clever, affable and courteous, but an impression is left with the visitor that something is expected in return. The Germans impress you by their brusqueness, at the same time with their kindness, for which no return is expected.

THE FRENCH AND THE GERMANS.

The French laugh much and mean little; the German laughs little and means much. The French are more polished and refined; the German has depth and solidarity which means more. The French say "au revoir, come again;" the German says "stay on with us." The French drink wine, the German beer; one scintillates, the other thinks. The French are small in stature, the German large. The French, even the women, have ugly complexions; the German clear skin and blue eyes. One is quick and vivacious, the other slow and steady. If the two countries are to be judged in a military way by the appearance of their respective armies, such at least as I have seen, then my sympathy goes out to the French in case of war, if appearances go for anything.

The French soldier is small, listless; strange to say, seems badly nourished and poorly drilled. The German soldier is large, square built, erect, walks with a steady step and looks well nourished. Do these two splendid Powers love each other? In the circle that goes to make up the Place de la Concorde there are numerous monuments erected in memory of lost provinces. Among them is one dedicated to Alsäce-Lorraine. This one is always seen enveloped in mourning habiliment. If you will ask a Frenchman why this is he will say: "We mourn her loss and keep her memory green until she is ours again." It is only by force of arms that this hope can be turned into a fruition. Should it ever come to this who would be the victor? Will not the result of the Franco-Prussian war suffice as an answer? No, they do not love one another.

Tomorrow I will take steamer at Mayence to go down the Rhine to Cologne, thence to The Hague. If I have time and you will accept, I will try and send some impressions of that very cosmopolitan town, the residence of the little, sweet-faced Dutch Queen of Holland, a town that is in reality "spotless clean," for are not the scrubbers always scrubbing? J. M. Mathews, M. D.

Wiesbaden, Germany, July 15, 1909.

